# Study of Hygrothermal Aging for Basalt Fiber/Epoxy Resin Composites Modified with CeCl_3_

**DOI:** 10.3390/polym16060819

**Published:** 2024-03-15

**Authors:** Chong Li, Longwang Zhang, Haoyu Wang, Yiguo Song, Jiayou Wang

**Affiliations:** 1 College of Mechanical and Electrical Engineering, Harbin Engineering University, Harbin 150001, China; songyiguo@hrbeu.edu.cn; 2 College of Materials Science and Chemical Engineering, Harbin Engineering University, Harbin 150001, China; zhanglongwang2022@163.com (L.Z.); haoyuwang@hrbeu.edu.cn (H.W.); wangjiayou163163@163.com (J.W.)

**Keywords:** rare earth modification, composites, basalt fibers, hygrothermal aging

## Abstract

With increasing attention being paid to environmental issues, the application of natural fibers in fiber-reinforced composites has attracted more and more attention. Composite materials with basalt fibers (BFs) as reinforcement have excellent properties and are widely used in many fields. Hydrothermal aging crucially influences the durability of basalt fiber/epoxy resin composites (BF/ERCs). In this study, BFs were used as reinforcing materials, whose surfaces were modified with a rare earth modification solution (CeCl_3_). The density, mechanical performance, and chemical properties of BF/ERCs subjected to hygrothermal aging were analyzed by the weight method, static mechanical performance testing, scanning electron microscopy (SEM), and Fourier transform infrared spectroscopy (FT-IR). The effects of the modification solution with different Ce concentrations on the water absorption, tensile, bending and interlaminar shear strength (ILSS) of BF/ERCs were investigated. The test results showed that the water absorption of BF/ERCs treated with a modification solution that contained Ce 0.5 wt % as the minimum value and the retention rate of the mechanical properties of BF/ERCs reached maximum values after hygrothermal aging.

## 1. Introduction

Fiber-reinforced polymer composites (FRPCs) are widely used in electrical engineering, aerospace, marine, energy applications and other fields, because of their excellent properties such as high specific strength, high specific modulus, corrosion resistance and fatigue resistance. In the service process of composites, the surrounding environmental factors including high/low temperature, moisture, ultraviolet light, oxidation and cyclic loading will reduce the performance of the material. The degradation of various mechanical properties of composites limits the application of FRPCs [1,2,3,4,5,6,7]. In particular, the thermal/wet conditions are the important application conditions of FRPCs in infrastructure, the food industry, vehicles and ships, which may lead to the deterioration of the moisture absorption and mechanical properties of FRPCs, that is, hygrothermal aging. This is considered to be a hot spot in aging research regarding composites [8].

In the hygrothermal environment, the matrix and the interface between fibers and the matrix are the main factors affecting the overall mechanical properties of FRPCs. The hygrothermal environment will cause the swelling of the resin matrix, reducing the dimensional stability, strength and stiffness of the composite, thus affecting the performance of the composite during service and its final service life. The epoxy system may degrade in the hygrothermal environment. In addition to the typical failure modes such as matrix cracking, fiber breakage, debonding and delamination, the adsorbed water may also cause early failure through various processes such as pitting, hydroxylation, hydrolysis, plasticization and leaching. The strength of the interface between the fiber reinforcement and the resin matrix in the composite has an important influence on the performance of the material after aging. The interfacial bonding performance between the fiber and the matrix decreases, and the macroscopic mechanical properties will decrease. In the static and fatigue failure of composites after aging, interface damage is often the main damage mode. Therefore, it is necessary to improve the performance of a composite after aging by improving the interfacial bonding performance of the composite, to test whether the performance of the composite meets the design requirements by simulating the hygrothermal environment in the laboratory and to further study the hygrothermal aging mechanism of the composite [8,9,10,11,12,13].

In recent years, with increasing attention being paid to environmental issues, the application of natural fibers in FRPCs has attracted more and more attention. BFs are formed from basalt rocks using conventional equipment through the melting and drawing process, and the preparation process does not cause environmental pollution. At the end of service, BFs can be degraded in soil to minimize the pollution to the environment. BFs have good mechanical properties, outstanding thermal stability, corrosion resistance, ideal thermal insulation, sound absorption and low water absorption and low cost. They have replaced or partially replaced other fibers in some fields as reinforcements in composites and have broad market prospects in the fields of fire protection, environmental protection, aerospace, the automobile and shipbuilding industry and construction [14,15,16,17,18,19,20].

BFs have many advantages in terms of performance, but the surfaces of BFs are chemically inert and cannot be fully integrated with the resin matrix during the preparation of composites, resulting in low interfacial adhesion between BFs and the matrix, which limits the industrial application of BF-reinforced composites. Therefore, it is necessary to carry out the surface chemical or physical modification of BFs [21,22,23]. Through modification treatment, not only the surface roughness of the fiber can be increased to make the fiber surface more suitable for bonding with the resin matrix, but also, the active groups on the fiber surface can be increased to improve the chemical activity of the fiber surface, so as to improve the overall performance of the composite by improving the interfacial bonding strength.

There are many methods to modify the fiber surface, such as coupling agent modification, plasma modification, oxidation modification, coating modification, acid–base etching modification and so on. Fu H. J. et al. [24] modified the surface of BFs by a coupling agent and analyzed the mechanical properties of the modified composites. The results showed that compared with the composites before modification, the interfacial bonding properties of the composites modified by the coupling agent were greatly improved, and the ILSS measured by a mechanical test was about 24% higher than that before modification. Liu T. et al. [25] used the coupling agent KH-550 to improve the mechanical properties of BF-reinforced nylon 66 composites. M. T. Kim et al. [22] found that the surface roughness of BFs modified by low-temperature oxygen plasma was increased and the interface properties between the fiber and the resin matrix were improved, which thus improved the ability of the composite to resist external loads. The fracture toughness of the modified composite increased by 16% compared with that before modification. Cheng et al. [26,27,28] investigated the effect of surface treatment on the interfacial bonding properties between F-12 aramid fiber, carbon fiber and the matrix. They found that rare earth modification solution treatment can increase the concentration of active functional groups on the fiber surface through a chemical coordination reaction. Therefore, the interfacial bonding performance between the fiber and the matrix is improved significantly. The tensile property of the composites can be improved obviously. At the same time, the tensile strength of a single fiber is almost not affected after the treatment with rare-earth-modified solution. Treating BFs with the rare earth modification solution is an attractive method because it has the advantages of high efficiency, low cost, a simple process, no pollution to the environment and no damage to fibers. However, there are few studies on the rare earth modification of BFs and the hydrothermal aging properties of BF/ERCs treated with rare earth modification solution.

In this paper, BFs were used as reinforcing material, whose surfaces were treated with the rare earth modification solution. Then, by simulating a hot and humid environment in the laboratory, the properties of BF/ERCs not treated and treated with the modification solution were analyzed by the weight method, a static mechanical performance test, SEM and FT-IR analysis before and after hygrothermal aging. The effects of the modification solution with different Ce concentrations on the tensile, bending and ILSS properties of BF/ERCs were investigated. On the above foundation, the hygrothermal aging mechanism of composites and the modification mechanism of rare earth elements were discussed.

## 2. Materials and Methods

### 2.1. Materials

In the experiment, BF cloth was purchased from Shanxi Baseote Technology Co., Ltd. (Taiyuan, China) with the performance parameters shown in Table 1. The epoxy resin (LY564) and the aliphatic amine curing agent (22964) were provided by Huntsman Co., Ltd. (Salt Lake City, UT, USA). The other chemical reagents used in the experiment are shown in Table 2.

### 2.2. Sample Preparation

The components of the BF modification solution, cerium chloride, anhydrous ethanol, citric acid and urea, were uniformly mixed at a certain ratio. With the four cerium addition levels of 0.1 wt %, 0.3 wt %, 0.5 wt % and 0.7 wt %, four kinds of cerium modification solutions were prepared.

The BF cloth was cut into 260 mm × 30 mm segments and then cleaned by ultrasonic cleaning for 20 min to remove the impurities on the fiber surface. After being dried in a drying oven, the BF cloths were, respectively, immersed in four kinds of cerium modification solution at room temperature for 2 h. Then, the treated BF cloths were dried in a drying oven at 85 °C for 1 h, and the modified BF cloths were obtained.

The prepared BF cloths were immersed in epoxy resin/the curing agent at a volume ratio of 4:1. With the hand lay-up method, the BFs/ERCs were fabricated after being cured at 120 °C for 15 min and being cured at 140 °C for 2 h, respectively.

### 2.3. Experiment and Test

The hygrothermal aging of the samples was carried out in a distilled water bath at 95 °C. The initial weight of each sample was measured before hygrothermal aging. During the aging process, samples were taken out of the water bath every 2 h. The surface moisture was wiped with absorbent paper and the sample weight was measured, and then, they were quickly put back into the water bath again for further hygrothermal aging. The average weight of five samples was calculated. The water absorption w is defined as follows:

(1) $$w=\frac{M_{t}-M_{0}}{M_{0}}\times 100\%$$

where *M_t_* is the weight of the sample at time *t*; *M*_0_ is the initial weight of the sample.

After hygrothermal aging for 24 h, the change in chemical structure was analyzed by FT-IR spectroscopy (Perkin Elmer 100) for samples not treated and treated with the modification solution containing different concentrations of Ce.

Before and after hygrothermal aging, the tensile strength, bending strength and ILSS of samples not treated and treated with the modification solution containing different concentrations of Ce were determined by the universal material testing machine WOW-50 and complied strictly with the corresponding standard [29,30,31]. The test results were the average values of five samples for every test.

The tensile fracture surface morphology of samples after hygrothermal aging was observed by a scanning electron microscope (SEM) JSM-6480 (Japan Electron Optics Laboratory Co., Ltd., Tokyo, Japan). Data were collected at the accelerating voltage of 20 kV. Prior to the characterization of SEM, the samples were sputter-coated with a thin layer of gold in vacuum to improve the electrical conductivity.

## 3. Results and Analyses

### 3.1. Water Absorption Characteristics of BF/ERCs

**Figure 1:**
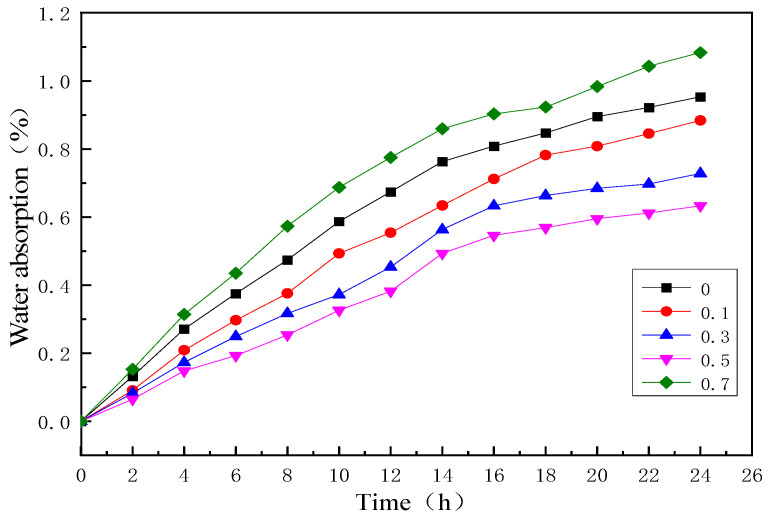
Water absorption curves of BF/ERCs.

The water absorption curves of BF/ERCs not treated and treated with the cerium-salt-modified solution are shown in Figure 1. The water absorption of BF/ERCs increased as the immersion time increased. The absorption process can be divided into two stages. In the first stage, the water absorption of BF/ERCs not treated and treated with the cerium-salt-modified solution increased almost linearly with the immersing time, which was in the stage of rapid water absorption. In the second stage, the water absorption increased slowly and tended to be stable.

Moisture in the exterior can enter the interior of a composite material through three ways: (1) the capillary action between the interface of the fiber and the resin matrix; (2) diffusion through the resin matrix; (3) entry through various defects in the composite material, such as tiny cracks and holes in the interface layer. In the first stage, water molecules invade the surface of the composite sample and occupy the free volume in the tiny pores inside the material. With the increase in hygrothermal aging time, the water absorption of the composites increases. In the second stage, water molecules begin to enter the interior of the composite sample. The polymer chain of the epoxy resin matrix is slowly rearranged due to the penetration of water molecules, resulting in free volume among polymer molecules and further moisture absorption. However, the relaxation rate of polymer molecules is significantly smaller than the diffusion rate of water molecules, so the moisture absorption increases slowly.

It can be seen that the water absorption of BF/ERCs treated with the cerium-salt-modified solution decreased with the Ce concentration increasing from 0.1 wt % to 0.5 wt %, and all water absorption levels were lower than those of untreated BF/ERCs. When the modification solution contained Ce 0.5 wt %, the water absorption of BF/ERCs was lowest, and the water absorption was 0.633% after hygrothermal aging for 24 h.

When the Ce content exceeded 0.5 wt %, the water absorption of BF/ERCs increased with the increase in Ce content. The water absorption of BF/ERCs increased to the highest level, 1.038%, when the Ce concentrations increased to 0.7 wt %.

### 3.2. FT-IR Spectra of BF/ERCs after Hygrothermal Aging

**Figure 2:**
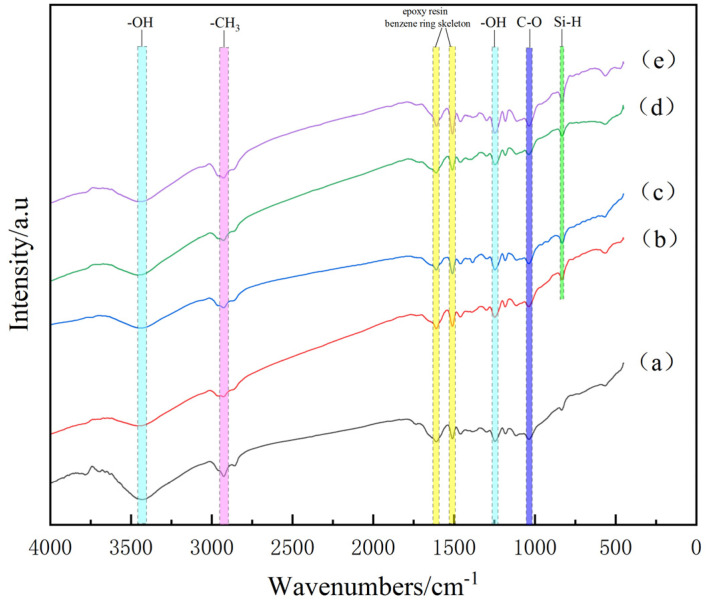
FT-IR spectra of BF/ERCs after hygrothermal aging:(a) untreated; (b) treated with modification solution containing Ce 0.1 wt %; (c) treated with modification solution containing Ce 0.3 wt %; (d) treated with modification solution containing Ce 0.5 wt %; (e) treated with modification solution containing Ce 0.7 wt %.

The chemical structure of BF/ERCs not treated and treated with the cerium-salt-modified solution after hygrothermal aging was analyzed by FT-IR spectroscopy, as shown in Figure 2. It can be seen that the infrared spectra of the BF/ERCs not treated and treated with different concentrations of cerium-salt-modified solution were basically similar after hygrothermal aging, but the intensity of the absorption peak changed. This indicates that no new substance was produced in the composite after hygrothermal aging. The characteristic peaks of -OH at 3430 cm^−1^ and 1249 cm^−1^, -CH_3_ at 2920 cm^−1^ and C-O at 1182 cm^−1^ occurred. The absorption peaks of the epoxy resin benzene ring skeleton were at about 1608 cm^−1^ and 1510 cm^−1^. The characteristic peak at 828 cm^−1^ is related to Si-H, which only occurred on the infrared spectrum of BF/ERCs modified with the cerium salt solution.

By comparison, it could be found that the intensity of the absorption peak of the active group of BF/ERCs modified with the cerium salt solution was higher than that of the untreated BF/ERCs on the infrared spectrum after hygrothermal aging. This indicates that more active groups were brought to the surface of BFs by rare earth elements, so that the interfacial bonding strength of the composites increased. According to the number and shape of the characteristic peaks in Figure 2, it can be concluded that when the Ce concentration in the modified solution reached 0.5 wt %, there were the most active groups of the composite material.

### 3.3. Mechanical Properties of BF/ERCs

**Figure 3:**
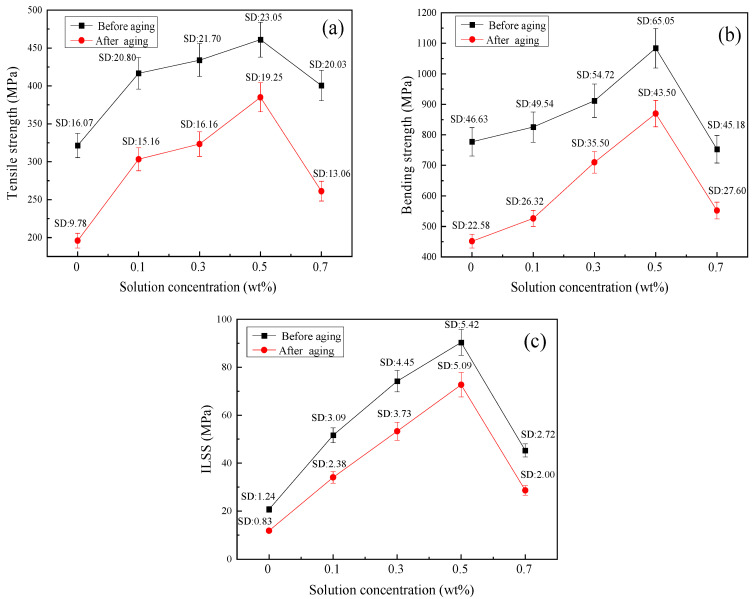
Mechanical properties of BF/ERCs: (**a**) tensile strength; (**b**) bending strength; (**c**) ILSS.

Tensile, bending and ILSS tests were performed for BF/ERCs before and after hygrothermal aging. The experimental data are shown in Figure 3. Compared with untreated BF/ERCs, the mechanical properties such as the tensile strength, bending strength and ILSS of BF/ERCs treated with the cerium salt modification solution were all improved. With the increase in Ce content from 0.1 wt % to 0.5 wt %, the mechanical properties of BF/ERCs before and after hygrothermal aging increased to the maximum value. When the Ce content exceeded 0.5 wt %, the mechanical properties of BF/ERCs decreased with the increase in Ce content.

Before hygrothermal aging, when the Ce content was 0.5 wt %, the tensile strength, bending strength and ILSS of BF/ERCs reached the maximum values, which were 460.99 MPa, 1084.13 MPa and 90.34 MPa, respectively. They were 43.4%, 39.5% and 336.2% higher than those of unmodified BF/ERCs.

**Figure 4:**
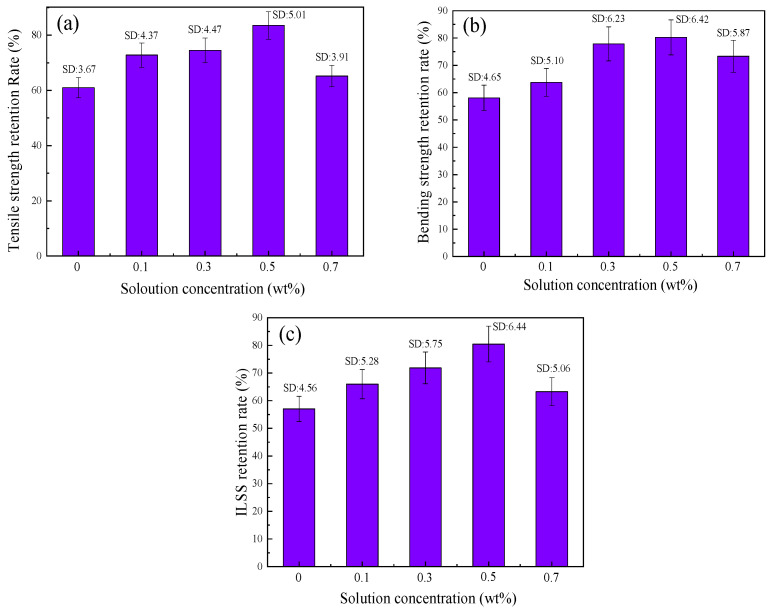
Retention rate of mechanical properties of BF/ERCs after hygrothermal aging: (**a**) retention rate of tensile strength; (**b**) retention rate of bending strength; (**c**) retention rate of ILSS.

After hygrothermal aging at 95 °C for 24 h, the mechanical properties of BF/ERCs not treated and treated with cerium-salt-modified solution all decreased, and a histogram of the retention rate of mechanical properties is shown in Figure 4. It can be seen that the retention rate of the mechanical properties of BF/ERCs treated with the cerium salt modification solution was improved. With the increase in Ce content from 0.1 wt % to 0.5 wt %, the retention rate of the mechanical properties of BF/ERCs increased to the peak value, and the retention rates of tensile strength, bending strength and ILSS were 83.51%, 80.25% and 80.48%, respectively. When the Ce content was more than 0.5 wt %, the retention rate of mechanical properties of BF/ERCs decreased with the increase in Ce content.

### 3.4. Fracture Surfaces Morphology of BF/ERCs after Hygrothermal Aging

**Figure 5:**
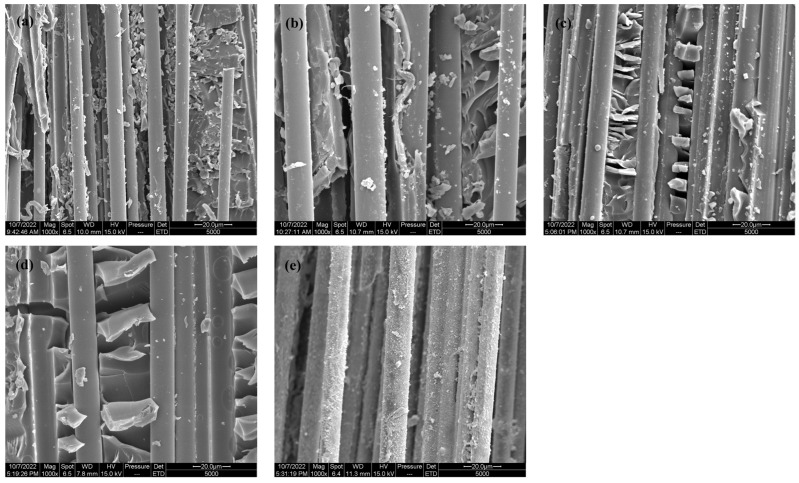
SEM images of tensile fracture surfaces of BF/ERCs after hygrothermal aging: (**a**) untreated; (**b**) treated with modification solution containing Ce 0.1 wt %; (**c**) treated with modification solution containing Ce 0.3 wt %; (**d**) treated with modification solution containing Ce 0.5 wt %; (**e**) treated with modification solution containing Ce 0.7 wt %.

Figure 5 shows the tensile fracture morphology of BF/ERCs not treated and treated with cerium-salt-modified solution after hygrothermal aging. As shown in Figure 5a, smooth fibers and grooves could be seen on the fracture surfaces of untreated BF/ERCs, and little resin was adhered on the surface of fibers. Some fibers were broken and pulled out from the matrix. It was shown that the bad interfacial adhesion of untreated BF/ERCs led to the great ingress of water. The water absorption rate accelerated. The damage of BF/ERCs under the external load mostly occurred at the interface between the fiber and the resin matrix, so the mechanical properties of BF/ERCs after hygrothermal aging decreased obviously.

Figure 5b,c show that the residual resin matrix on the fracture surface increased with an increasing Ce concentration. This indicated that the interfacial adhesion between the fiber and the resin matrix was improved and the water absorption of BF/ERCs was controlled effectively, which reduced the erosion of water into BF/ERCs. After hygrothermal aging, the interface between the fiber and the resin matrix could bear a greater external load, the anti-aging performance of BF/ERCs was improved. The mechanical properties test results showed that the retention rate of the mechanical properties of BF/ERCs increased.

When the Ce concentration was 0.5 wt %, there was more resin on the surface of fibers, and no fibers were broken or pulled out from the matrix, as shown in Figure 5d. The interfacial adhesion between the fiber and the resin matrix was best, and the water absorption of composites was worst. After hygrothermal aging, the retention rate of the mechanical properties of BF/ERCs was at its maximum value.

When the Ce concentration was up to 0.7 wt %, the resin gathered into blocks on the fiber surfaces, as shown in Figure 5e. The nonuniform microstructure at the interface caused the bad interfacial adhesion between the fiber and the resin matrix and delamination between fibers. The stress that the interface could transfer was reduced. After hygrothermal aging, the moisture absorption rate of BF/ERCs increased, and the retention rate of the mechanical properties decreased.

### 3.5. Hygrothermal Aging Properties of BF/ERCs

In the hygrothermal environment, water is mainly absorbed by the resin matrix, and BFs are basically non-hygroscopic, resulting in the difference in volume expansion between BFs and the resin matrix, which causes cracking and debonding at the interface. In addition, the entry of water produces osmotic pressure inside the composite, which causes cracks inside the resin matrix. The generation of defects such as cracks and pores makes the entry of water molecules easier. If the hygrothermal aging time is too long, the resin matrix can be hydrolyzed, resulting in the fracture and decrosslinking of the polymer chain. These aggravate the damage of the interface between fibers and the resin matrix, thus reducing the mechanical properties of the composite [32]. The faster the water absorption rate, the greater the decrease in the mechanical properties of BF/ERCs.

For BF/ERCs treated with the cerium salt modification solution, rare earth elements can make more oxygen-containing active functional groups attach to the surface of BFs. In addition, rare earth elements are used as an intermediate medium to coordinate and bond with the active groups on the surface of BFs and the organic groups in the epoxy resin and form a stable chemical bond connection, which can strengthen the interfacial bonding strength between BFs and the resin matrix. The mechanical properties of BF/ERCs are improved. During the process of hygrothermal aging, good interfacial bonding performance and few defects at the interface can hinder the diffusion of water molecules, control the water absorption rate effectively and reduce the destruction of the interface. After hygrothermal aging, the retention rate of the mechanical properties of BF/ERCs is high. The anti-aging performance of BF/ERCs treated with the appropriate concentration of cerium salt modification solution can be improved.

## 4. Conclusions

In this study, the effects of the Ce concentration in a rare earth modification solution on the water absorption and the mechanical properties of BF/ERCs were discussed after hygrothermal aging.

The Ce element affected the water absorption of BF/ERCs treated with the rare earth modification solution. When the modification solution contained a Ce concentration of 0.5 wt %, the water absorption of BF/ERCs was lowest and 24 h water absorption was 0.633%. The tensile strength, bending strength and ILSS of BF/ERCs treated with the modification solution containing a Ce concentration of 0.5 wt % reached the maximum value before and after hygrothermal aging. The retention rate of the mechanical properties of BF/ERCs treated with the rare earth modification solution containing Ce 0.5 wt % were up to the peak values. The retention rates of the tensile strength, bending strength and ILSS of composites all exceeded 80% after hygrothermal aging. Therefore, the hydrothermal aging resistance of BF/ERCs treated with the rare earth modification solution can be improved. The suitable Ce content is 0.5 wt %.

## Figures and Tables

**Table 1:** Performance parameters of BF cloth.

Diameter (μm)	Density (g/cm^3^)	Line Density (g/km)	Water Content (%)	Tensile Strength (MPa)	Elastic Modulus (GPa)	Breaking Elongation (%)
5.9	2.7	223	0.1	1835	7.5	2.75

**Table 2:** Chemical reagents in the experiment.

Chemical Reagent	Molecular Formula	Purity	Producer
Anhydrous ethanol	C_2_H_6_O	Analytically pure	Tianjin Tianda Chemical Reagent Factory, Tianjin, China
Citric acid	C_6_H_8_O_7_	Analytically pure	Tianjin Tianli Chemical Reagent Co., Ltd., Tianjin, China
Urea	CO(NH_2_)_2_	Analytically pure	Tianjin Zhiyuan Chemical Reagent Co., Ltd., Tianjin, China
Cerium chloride	CeCl_3_·6H_2_O	99.99%	Jining Zhongkai New Type Material Co., Ltd., Tianjin, China

## Data Availability

The data presented in this study are available on request from the corresponding author. The data are not publicly available as the data are part of an ongoing study.
